# Full mitochondrial genome sequences of two endemic Philippine hornbill species (Aves: Bucerotidae) provide evidence for pervasive mitochondrial DNA recombination

**DOI:** 10.1186/1471-2164-12-35

**Published:** 2011-01-14

**Authors:** Svenja Sammler, Christoph Bleidorn, Ralph Tiedemann

**Affiliations:** 1 University of Potsdam, Institute for Biology and Biochemistry, Unit of Evolutionary Biology/Systematic Zoology, Karl-Liebknecht-Str. 24-25, Haus 26, D-14476 Potsdam, Germany; 2 University of Leipzig, Institute for Biology II, Molecular Evolution and Systematics of Animals, Talstr. 33, D-04103 Leipzig, Germany

## Abstract

**Background:**

Although nowaday it is broadly accepted that mitochondrial DNA (mtDNA) may undergo recombination, the frequency of such recombination remains controversial. Its estimation is not straightforward, as recombination under homoplasmy (i.e., among identical mt genomes) is likely to be overlooked. In species with tandem duplications of large mtDNA fragments the detection of recombination can be facilitated, as it can lead to gene conversion among duplicates. Although the mechanisms for concerted evolution in mtDNA are not fully understood yet, recombination rates have been estimated from "one per speciation event" down to 850 years or even "during every replication cycle".

**Results:**

Here we present the first complete mt genome of the avian family Bucerotidae, i.e., that of two Philippine hornbills, *Aceros waldeni *and *Penelopides panini*. The mt genomes are characterized by a tandemly duplicated region encompassing part of *cytochrome b*, 3 tRNAs, *NADH6*, and the control region. The duplicated fragments are identical to each other except for a short section in domain I and for the length of repeat motifs in domain III of the control region. Due to the heteroplasmy with regard to the number of these repeat motifs, there is some size variation in both genomes; with around 21,657 bp (*A. waldeni*) and 22,737 bp (*P. panini*), they significantly exceed the hitherto longest known avian mt genomes, that of the albatrosses. We discovered concerted evolution between the duplicated fragments within individuals. The existence of differences between individuals in coding genes as well as in the control region, which are maintained between duplicates, indicates that recombination apparently occurs frequently, i.e., in every generation.

**Conclusions:**

The homogenised duplicates are interspersed by a short fragment which shows no sign of recombination. We hypothesize that this region corresponds to the so-called Replication Fork Barrier (RFB), which has been described from the chicken mitochondrial genome. As this RFB is supposed to halt replication, it offers a potential mechanistic explanation for frequent recombination in mitochondrial genomes.

## Background

Since Desjardins and Morais [1] have presented the mt gene organization of the domestic chicken (*Gallus gallus*), it is known that birds possess a different gene order compared to other vertebrates. While the chicken gene order was found in many other avian taxa as well, Mindell et al. [2], Eberhard et al. [3], Abbott et al. [4], and Verkuil et al. [5] subsequently presented alternate avian mt gene orders and discussed their potential origin. Gibb et al. [6] suggested a conversion scenario for avian species according to the tandem duplication and random loss [TDRL] model [7,8]. Specifically, they assume the derived avian gene order to have originated from an initial tandem duplication of the *Cytb*/tRNA T/tRNA P/*NADH6*/tRNA E/CR region, followed by several gene losses or reductions. Up to now, the completely conserved tandem duplicate is only reported for albatrosses [4,6], spoonbills [9], and boobies [10]. Although further intermediate forms with two apparently functional gene or control region (CR) duplicates are rarely found (but see [3,5,6,11,12]), it is generally assumed that the derived gene order has evolved independently more than once [2].

Studying mantellid frogs from Madagascar, Kurabayashi et al. [13] infer two other possible mechanisms of mt genome reorganization than the TDRL model, both duplication modes mediated by recombination. One mechanism is the "illegitimate recombination via minicircle" (e.g., [14,15]), where one part of the mt gene region is excised from one mt genome, forming a separate minicircle molecule. This molecule is then inserted into another genome, resulting in nontandem-duplicated regions within the mtDNA molecules. Another mechanism is the "general (homologous) recombination" (e.g., [16,17]), where DNA strands of two genomic portions with identical or similar nucleotide sequences between chromosomes or within a DNA molecule are exchanged. When the exchanged DNA strands contain the same set of genes or regions, this recombination process does not cause gene duplication but can homogenize the sequences (gene conversion). On the contrary, when the exchanged DNA strands carry unequal sets of genes, one of the resultant molecules or genomic portions will have an extracopied gene region (unequal crossing over).

Recombination of the putative clonally maternally inherited mitochondrial DNA has been detected in several animal species, including birds and mammals [18]. The frequency of such recombination, however, remains controversial; its estimation is not straightforward, as recombination under homoplasmy (i.e., among identical mt genomes) is likely to be overlooked [19]. In species with tandem duplications of large mtDNA fragments such as in albatrosses [4], spoonbills [9] and boobies [10], the detection of recombination is possible, as it can lead to gene conversion among duplicates. Although the mechanisms for concerted evolution in mtDNA are not fully understood yet, recombination rates have been estimated from "one per speciation event" among albatross species [4], down to 850 years among populations of killifish [20]. The study of Ogoh and Ohmiya [21] even shows that gene conversion in the mt genome of ostracods occurs during every replication cycle. To explain their results, Ogoh and Ohmiya [21] suggest a different mechanism. According to them, an exact replication mechanism, not recombination, controls the concerted evolution: the duplicated fragment is deleted and duplicated afresh in every replication cycle.

Besides studies on recombination and on rearrangements of mt genes, mtDNA as such is considered as a valuable tool in population genetic, phylogeographic, and phylogenetic studies [22]. Because useful information can be detected from many of the mt genes and due to primers being functional for a wide range of avian taxa [23,24], the number of completely sequenced avian mt genomes is steadily increasing (e.g., [6,25-38]. Nevertheless, no mt genome from the family Bucerotidae is known so far and hornbills are missing from many phylogenetic analyses.

Here we present the complete mt genomes of two Philippine hornbills, endemic to the West Visayas, the Rufous-headed Hornbill *Aceros waldeni *and the Visayan Tarictic Hornbill *Penelopides panini*, and compare their characteristic mt genome features to each other and to those of other birds. We specifically test the hypothesis that recombination of mtDNA occurs regularly, i.e., within individuals of local animal populations.

## Results and discussion

### Genome organization

**Figure 1:**
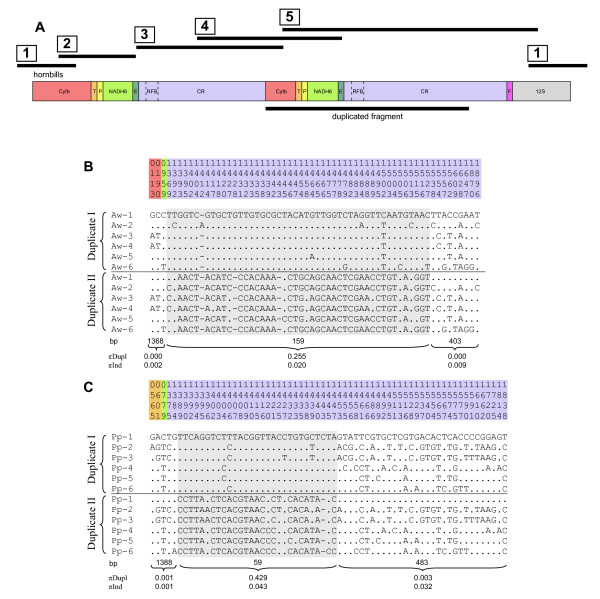
**mtDNA genome organization in Philippine Hornbills (only tandemly duplicated part) (A) Gene order and position of five overlapping PCR-amplificates. (B, C) Variable sites of 1,930 bp alignments of the duplicated fragments of *A. waldeni *(B) and *P. panini *(C)**. The grey shaded sections represent the putative Replication Fork Barrier (RFB) regions. πDupl is the average diversity among duplicates *within any individual*. πInd is diversity *among individuals *for the same duplicate (I or II).

The two new mt genome sequences of the Philippine hornbills have been deposited in NCBI GenBank under the accession numbers HQ834450 (*A. waldeni*) and HQ834451 (*P. panini*). As expected and known from other birds, *NADH6 *and 8 tRNAs are transcribed from the light strand. All other 12 protein coding genes, 14 tRNAs and the two rRNAs *12S *and *16S *are located on the heavy strand (see Additional file 1: Table S1. Sequence annotation of the mt genome of *A. waldeni*/*P. panini *(as in deposited sequence)). The mt genomes of the two hornbills are longer than any other avian mt genome reported so far. The deposited sequences are 21,657 bp in *A. waldeni *and 22,737 bp in *P. panini*, both substantially exceeding the hitherto longest known avian mt genome of *Diomedea melanophris *(18,967 bp) [6]. The gene order is characterized by a tandemly duplicated region beginning with the last 526 bp of *Cytb*, continuing over tRNA T/tRNA P/*NADH6*/tRNA E, and ending after CRII (Figure 1A). The final length of the mt genome is the result of this duplication event, a repeat motif with remarkably long units found in both CRs, and another tandem repeat at the end of CRII. As there was some intraindividual variation (heteroplasmy) in the number of these repeats, total mt genomic lengths of both hornbill species were variable (see below).

### Structure of the duplicated region

**Figure 2:**
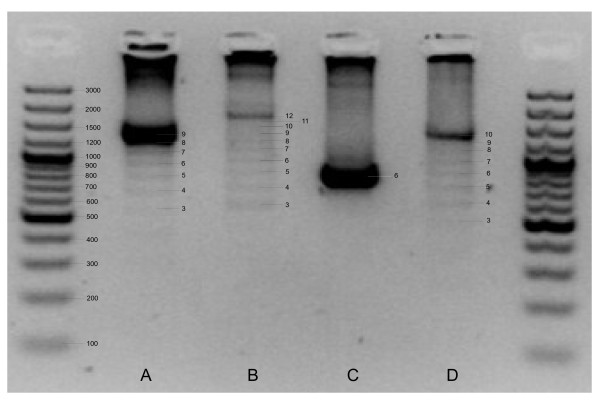
**Nested PCR-amplificates spanning over the repetitive units in domain III of the control region**. Number of repeat units is indicated by the scale superimposed over the amplificates. (A) CRI of *A. waldeni *(dominant: 9 repeats). (B) CRI of *P. panini *(dominant: 12 repeats). (C) CRII of *A. waldeni *(dominant: 6 repeats). (D) CRII of *P. panini *(dominant: 10 repeats).

**Figure 3:**
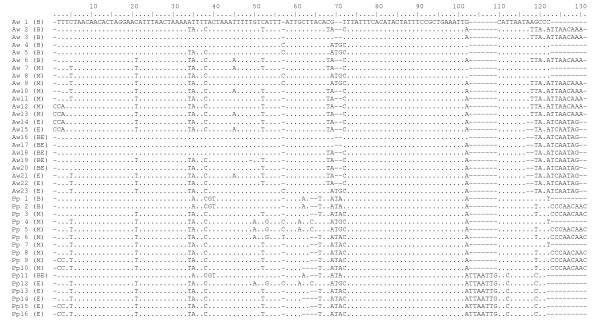
**Repeat units found in CRI und CRII of *A. waldeni *(Aw) and *P. panini *(Pp)**. B: found only at the beginning. M: found only between B and E. E: found only at the end. BE: repeat unit of clones with only one unit.

Within individuals, the duplicated fragments are identical for the first 1,368bp (*A. waldeni*) or 1,388 bp (*P. panini*), except for a single substitution in two specimens (position 565 (tRNA T) in Pp-1 and position 1384 (CR) in Pp-6; cf. Figure 1B, C). This region contains the duplicated part of *Cytb*, tRNA T, tRNA P, *NADH6*, and tRNA E, and the first part of domain I of the control regions (CR, Figure 1). In the following 159 bp of *A. waldeni*, the duplicated fragments greatly differ within each individual at a total of 48 polymorphic sites, including three indels such that CRII is 2-3 bp shorter than CRI (Figure 1B). In *P. panini*, the duplicates differ within individuals at 28 polymorphic sites and 1 indel position over a length of 59 bp (Figure 1C). In the remaining part of domain I and in the complete domain II (403 bp in *A. waldeni*, 483 bp in *P. panini*; defined from the start of the conserved F box to the start of the conserved sequence block (CSB) 1 according to the sequence of the chicken [1]), the duplicates are again identical within all analyzed specimens of *A. waldeni*, but exhibit a few variable sites in *P. panini *(Figure 1B, C). Domain III is characterized by a tandem repeat occuring in variable copy numbers, starting 37 bp (*A. waldeni*) or 27 bp (*P. panini*) after the end of the corresponding CSB1 of the domestic chicken. In the hornbills' CRI, the number of repeat units (determined by cloning and subsequent sequencing) varied between 1 and 10 (*A. waldeni*) or 1 and 17 (*P. panini*). The most common number was 9 units in *A. waldeni *and 12 units in *P. panini *(Figure 2A, B). In the total of 33 sequenced clones, 23 (*A. waldeni*) or 16 (*P. panini*) different repeat unit types between 111 bp and 123 bp in length were found. (Figure 3). The different repeat units did not occur in random order. Instead, repeat unit types were either (i) only found at the beginning and/or the end of the repeat region, or (ii) never occurred in that position, but only in between other repeats (Figure 3). Within each species, the first repeat unit always started with an identical motif of 19 bp (*A. waldeni*) or 98 bp (*P. panini*) (Figure 3). Likewise, the last repeat unit always ended with an identical motif of 18 bp (*A. waldeni*) or 49 bp (*P. panini*) (Figure 3). In CRII, the same types of repeat units were found. On average, CRII contains fewer repeat units than CRI. The number varies between 1 and 8 in *A. waldeni *(dominant 6, Figure 2C) and between 1 and 10 in *P. panini *(dominant 10, Figure 2D).

The following sequence of domain III (around 130 bp in *A. waldeni *and around 160 bp in *P. panini*) is identical among CRI and CRII within each of the species and exhibits at its beginning some similarity to the repeat motifs (data not shown; cf. to full mt genome sequences in Genbank accession numbers HQ834450 and HQ834451). In *A. waldeni*, CRI ends directly at the duplicated part of *Cytb*, whereas CRII continues after a spacer of 15 bp with another tandem repeat region. In *P. panini*, CRII passes directly into this second repeat region, whereas a spacer of 12 bp is situated between CRI and the duplicated part of *Cytb*. Concerning the second repeat region, the fully sequenced mt genome copies contained a truncated unit and between 1 and 27 (*A. waldeni*) or 33 (*P. panini*) complete units of 34 bp length. Even longer repeat regions (with presumably higher repeat numbers) existed, but sequence analysis did not reach through the entire repeat region of these very long mt genome variants, such that the exact number of repeats could not unambiguously be determined. The tandem repeat units exhibit a high A-content (47%) on the heavy strand. While *A. waldeni *possesses only one type of these tandem repeat units, different units with transitions at two sites are found in *P. panini*.

The organization of the mitochondrial genome found in the two hornbill species is most similar to that reported for albatrosses (*Thalassarche *spp. [4], *Diomedea melanophris *[6]), for the black-faced spoonbill [9], and for boobies [10]. It might constitute a general pattern at least in these two related taxonomic groups of seabirds (i.e., Procellariiformes and Pelecaniformes [6,10]).

Despite the general similarity in mitochondrial genome organization among albatrosses, the black-faced spoonbill, boobies, and hornbills, there are some fundamental differences among them: The last part of *Cytb*, with which the duplicated part begins, is significantly longer in hornbills, the spoonbill, and boobies, whereas in *Thalassarche *albatrosses this short *Cytb *part is preceded by a further part of *Cytb *considered to be degenerated [4].

Concerning CR domain III, the albatrosses and the black-faced spoonbill possess repeat motifs only in CRII, whereas in both hornbill species and in the three booby species studied by Morris-Pocock et al. [10], the first repeat motif is also found in CRI. Equally to the spoonbill study [9], neither Abbott et al. [4] nor Gibb et al. [6] mention length heteroplasmy in this motif of the albatross in their publications; however, in an updated version of the respective sequence of *Diomedea melanophris *albatross [GenBank: AY158677], heteroplasmy is assumed, as well as for the boobies [10]. Furthermore, confirmed length heteroplasmy in these motifs is described for other bird species, e.g., for the loggerhead shrike *Lanius ludovicianus *[39], for the little blue penguin *Eudyptula minor *[34], and for the ivory-billed aracari *Pteroglossus azara *[6]. Two sets of repeat motifs, as found in the studied hornbills in CRII, are also described for the control region of penguins (Adélie penguin, *Pygoscelis adeliae *[40] and little blue penguin [34]). The little blue penguin was found to be heteroplasmic for both of these motifs [34].

### Evidence for frequent recombination among mt genomes

**Figure 4:**
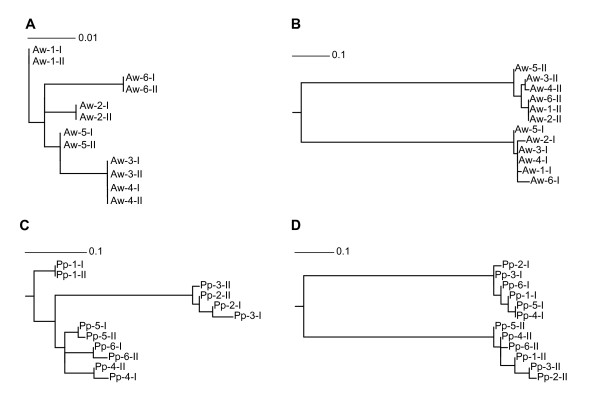
**Phylogenetic analyses for sections of the duplicated mt genome region**. ML, GTR+Γ+I-model of sequence evolution. Datasets are: (A) *A. waldeni*, section with high similarity between paralogues within individuals (=region of inferred recombination, white in Figure 1B) (B) *A. waldeni*, section with high similarity between orthologues across individuals (putative Replication Fork Barrier (RFB) region, grey in Figure 1B) (C) *P. panini*, region of inferred recombination (white in Figure 1C) (D) *P. panini*, putative RFB region (grey in Figure 1C). Roman letters (I, II) indicate CRI vs. CRII copies of single individuals. Aw, *Aceros waldeni*; **Pp**, *Penelopides panini*.

The analysis of the tandemly duplicated region in 6 individuals of each hornbill species enabled us to evaluate sequence evolution patterns across orthologous and paralogous duplicates over a total length of 1,930 bp, encompassing the duplicated parts of *Cytb*, tRNA T/tRNA P/*NADH6*/tRNA E and domains I and the first parts of domains II of the control regions (Figure 1, accession numbers HQ834450-HQ834471). This analysis revealed a remarkable shift in similarity pattern: In a central section (159 bp in *A. waldeni *and 59 bp in *P. panini*, grey shaded in Figure 1B, C), orthologous copies (duplicate I of all individuals and duplicate II of all individuals, respectively) are more closely related to one another across individuals than to paralogous copies (duplicate I and duplicate II) within individuals (*A. waldeni*: πInd = 0.020, πDupl = 0.255; *P. panini*: πInd = 0.043, πDupl = 0.429). This section is situated in domain I of the control regions. It is surrounded by sections with a reversed diversity pattern (1,368 bp and 403 bp in *A. waldeni*, 1,388 bp and 483 bp in *P. panini*), i.e., where paralogous copies within individuals are more closely related (and in fact fully identical for most specimens; πDupl between 0.000 and 0.003) than orthologous copies across specimens (πInd up to 0.032; Figure 1B, C). This striking shift in sequence similarity from similarity among orthologues across specimens (grey) to similarity/identity among paralogues within specimens (white) is also reflected in our sequence section-specific phylogenetic analyses using ML (Figure 4). These analyses suggest a homogenization among the duplicated fragments *within individuals *(Figure 4A, C), from which a distinct central sequence stretch (the grey shaded sections in Figure 1B, C) are exempted (Figure 4B, D). This homogenization encompasses the whole duplicated part of *Cytb*, tRNA T/tRNA P/*NADH6*/tRNA E, the first and last nucleotides of domain I and the sequenced part of domain II of the CR (altogether at least 1,771 bp in *A. waldeni *and 1,871 bp in *P. panini*). In this entire region, both duplicates within individuals are identical (except for one or two single base pair differences in a few specimens of *P. panini *(one synonymous Single Nucleotide Polymorphism (SNP) in tRNA T, the others in the CR); cf. Figure 1C). At the same time, there is sequence variation at orthologues among individuals, both in coding genes (one synonymous and one non-synonymous SNP in *Cytb *and one synonymous SNP in *NADH6 *of *A. waldeni*; one synonymous SNP in tRNA T and one synonymous SNP in *NADH6 *of *P. panini*) and in the control region of both species. If we compare any pair of specimens, their sequences in this region differ from one another at exactly the same nucleotide sites in both duplicates. This pattern indicates that the homogenization process must occur frequently, as it appears to have occurred in every single mitochondrial lineage within both species.

Kurabayashi et al. [13] postulated a novel scheme for vertebrate mtDNA replication, which can explain high frequencies of recombination. Applying this model to birds, replication of avian mtDNA is initiated throughout the mt genome, excluding the Replication Fork Barrier (RFB) [41]. During each replication cycle, the 3' end of the nascent L-strand is suspected to remain free at the RFB region until replication restarts. During the relatively long time of exposure, the free strands can easily be exchanged, leading to a high rate of recombination. This exchange can take place between two mtDNA molecules, but also within a single molecule (intragenomic gene conversion) if two independent replication forks occur.

For both hornbill species, the homogenization of the duplicates within individuals may be well explained with this recombination model. The part of the control region without intra-individual homogenization across duplicates (grey-shaded in Figure 1B, C; see above) putatively represents the RFB. Kurabayashi et al. [13] found recombination only on one side of the RFB. Our observation of homogenized sections on both sides of the putative RFB in hornbills (as also described for albatrosses [4], the black-faced spoonbill [9], boobies [10], and for the ruff [5]) may be explained by the fact, that replication occurs in both directions around the circular mtDNA in birds [41], with a prominent initiation zone between *Cytb *and *12S*, i.e., exactly in the genomic region duplicated in hornbills, albatrosses, the black-faced spoonbill, boobies, and the ruff.

For the CR of mammals, two types of so-called Extended Termination Associated Sequences (ETAS) have been described [42]. It is suggested that ETAS1 could contain recognition signals (primary and secondary structural elements) for the termination of the nascent DNA or RNA chain, while ETAS2 could contain the binding sites for termination factors [42]. Subsequently, ETAS were also found in birds (e.g., [43]). If we align the consensus sequence of mammalian ETAS1 and ETAS2 to our mt genome data of hornbills, they best match within the CR of the hornbills exactly in front of the putative RFB (i.e., exactly adjacent upstream to the grey-shaded region in Figure 1B, C). Putatively inferring this to be an ETAS region would further explain the exclusion of the inferred RFB from the recombination process.

### Organization of the mt genome in hornbills compared to other birds

Our complete sequences of the mitochondrial DNA of two hornbill species reveal many peculiar features in these mt genomes. Each of these features has been occasionally detected in a few avian taxa, but hornbills exhibit a unique combination in one single mt genome, i.e., (1) a tandem duplication of a region spanning over three tRNAs, one partial and one complete protein-coding gene, and the control region, (2) the existence of two sets of CR repeat motifs, of which one is duplicated as well, and (3) the remarkably long single units of these motifs, altogether making hornbills' mt genome with 21,657 bp (*A. waldeni*) and 22,737 bp (*P. panini*) the longest mt genome known from birds so far.

The hornbills' gene order differs from the gene order of most other avian taxa, except for two related groups of seabirds (Procellariiformes, Pelecaniformes [4,6,10]) and for the black-faced spoonbill (Ciconiiformes [9], assumed to be related to Procellariiformes and Pelecaniformes [44]). It has been named "duplicate tThr-CR" [6]. Because Procellariiformes/Pelecaniformes/Ciconiiformes and hornbills are generally not assumed to be sister taxa [44,45], our study indicates the independent evolution of the rare "duplicate tThr-CR" gene order. This gene order has been assumed to constitute an intermediate form between the "ancestral avian" and the "remnant CR(2)" gene orders, which is characterized by two apparently functional control region duplicates [6,11].

None of the peculiar features found in both Philippine hornbills was found in the control region of African hornbills - the closest relatives for which the CR has been examined [46]. Instead, the authors assumed the typical avian gene order ("ancestral type") for *Bucorvus leadbeateri *and several species of the genus *Tockus*. However, in the light of our results this assumption has to be re-examined. Delport et al. [46] sequenced only part of the mt genome (between tRNAs T and F), thus a duplication event might have been overlooked. Concerning further studies on the CR, we suggest for all avian taxa to sequence at least once the complete fragment between the end of *NADH5 *and the beginning of *12S *because all duplication events known so far have been located in this section.

Whereas repeat motifs are found in domain III of the Philippine hornbills, Delport [46] discovered such a motif in domain I of all African hornbills examined. Thus, an alignment of the CRs of the Philippine with those of the African hornbills was only possible for the conserved central domain II. In a phylogenetic analysis of this central domain, the two Philippine hornbills cluster together and are sister to the African species (see Additional file 2: Figure S1. ML-analysis of the central domain of the control region of Bucerotidae). In this data set, however, the authenticity of the published sequence of *Bucorvus leadbeateri *is rendered questionable, as it is indistinguishable from *Tockus erythrorhynchus*, despite the fact that *Bucorvus *and *Tockus *species are morphologically clearly apart [47]. Furthermore, the assumption that the repeat motif in domain I of the African hornbills had arisen before the adaptive radiation of all hornbill species, but after the divergence of hornbills from other avian taxa [46] clearly has to be rejected, as (1) Philippine hornbills do not show this sequence pattern and (2) our re-analysis of the data on African hornbills strongly suggest that Delport et al. [46] studied only members of the genus *Tockus*.

## Conclusion

Our full mt genome analysis of hornbill species revealed a large tandem duplication. Sequences within individuals are homogenized, except for the central putative Replication Fork Barrier. This sequence pattern suggests very frequent recombination of the mitochondrial genome. The studies of Eberhard et al. [3] and Ogoh and Ohmiya [21] were the first on concerted evolution in mitochondrial genomes in single species. In the ostracod *Vargula hilgendorfii*, Ogoh and Ohmiya [21] detected frequent gene conversion and suggested repeated deletion and exact duplication in every replication cycle as the underlying mechanism. For hornbills, this mechanism is very unlikely, as it would imply that deletion and exact duplication occur synchronously on both sides of the RFB in every replication cycle. In a study on mtDNA of mangrove killifishes, Tatarenkov and Avise [20] found indications for frequent recombination, but did not present any underlying molecular mechanism. In addition, they - as others - detected recombination specifically in the control region. For the case of Philippine hornbills, we present evidence for frequent gene conversion by recombination of a large section of the mitochondrial genome, encompassing several coding genes. Morris-Pocock et al. [10] sequenced only one individual of each booby species, but they also assume that more than the CR, namely part of *Cytb*/tRNA T/tRNA P/*NADH6*/tRNA E, evolve in concert. A possible mechanism is the existence of a Replication Fork Barrier, where mt genome replication is halted such that the 3' end of the replicated strand remains free and might hence easily recombine [13]. While there is no reason to assume that this mechanism is restricted to those species with a duplication in their mt genome, we argue that such duplication greatly facilitates our ability to unravel recombination: Without a duplication, recombination might affect orthologues and might hence go undetected. The duplication creates the additional possibility of intraindividual recombination among paralogues sequence parts. Such recombination causes a homogenization among these paralogues, clearly differing from the expectation of independent evolution after gene duplication.

## Methods

### Sampling and DNA extraction

One drop of blood per sample was taken from captive hornbills kept by the Philippine Endemic Species Conservation Project (PESCP) on Panay, Philippines, and stored in 1 ml Queen's Lysis Buffer [48]. DNA extraction was performed using the DNeasy Tissue Kit (Qiagen, Hilden, Germany) according to the manufacturer's instructions for blood samples. The two genomes presented here were sequenced using DNA from two single individuals. To test the hypothesis that recombination occurs within individuals, five further unrelated individuals of each species were sampled.

The research followed internationally recognized guidelines and applicable national legislation. We received ethical approval from the deputy of animal welfare of the University of Potsdam.

### Amplification, sequencing and cloning

To minimize the possibility of obtaining nuclear copies of mt genes, we amplified longer fragments starting by using the primers Pen_Cyt1065-for and Buce_12S240-rev (~4.5 kb) designed from published sequences of *Penelopides *spp. and other Bucerotidae and related taxa from NCBI databank (see Additional file 3: Table S2. PCR primers used to amplify and sequence mt gene fragments). The PCRs were performed using a long-range polymerase (LA Taq™, TaKaRa Bio Inc, Shiga, Japan). 15 μl-reaction volumes were set up as follows: 7.5 μl sterilized distilled water, 1.5 μl 10 x LA PCR™ Buffer II (Mg^2+^free), 1.5 μl 25 mM MgCl_2_-solution, 2.4 μl dNTP Mixture (2.5 mM each), 0.5 μl of each primer (2 mM), 1 μl DNA template (~20 ng/μl), 0.08 μl TaKaRa LA Taq™ (5 u/μl). The reaction was performed under the following conditions: denaturing at 94°C for 1 min, followed by 30 cycles of denaturing at 98°C for 10 s and elongating at 70°C for 4 min (without additional primer annealing step), and finally, an extended elongation period of 10 min at 72°C. The EXO-AP purified product was sequenced directly with PCR primers and additionally with internally primers designed by primer walking (see fragment 5 in Additional file 3: Table S2. PCR primers used to amplify and sequence mt gene fragments). The sequencing reactions were performed using BigDye Terminator Cycle Sequencing reagents version 3.1 and run on an ABI Prism 3130 Genetic Analyzer (Applied Biosystems, Foster City, CA, USA) according to the manufacturer's instructions.

To test for the existence of a duplicated control region we amplified a product with primers AcePen_KRII-for and AcePenGlu-rev (conditions as above, but 56°C annealing temperature for 20 s and 68°C elongating temperature for 3 min). This product was sequenced with PCR primers and internal primers (see Figure 1 and fragment 4 in Additional file 3: Table S2. PCR primers used to amplify and sequence mt gene fragments).

Due to the detected duplication event, we amplified the complete mt genomes in five overlapping fragments (Figure 1A), using the following primer combinations:

1. ~14 kb: AcePen_12S_68-for and AcePen_Cytb250-rev/AcePen_Cyt1018-rev (annealing and elongation both at 68°C for 14 min together);

2. ~1.6 kb: AcePen_Cytb253-for and AcePenGlu-rev (annealing at 60°C, elongation at 68°C for 1.5 min);

3. ~3.2 kb (*A. waldeni*), ~3,6 kb (*P. panini*): AcePen_Glu-for and AcePen_Cyt1018-rev (annealing at 60°C, elongation at 68°C for 3 min);

4. ~2.8 kb (*A. waldeni*), ~3,1 kb (*P. panini*): AcePen_KRII-for and AcePen_Glu-rev (annealing at 56°C, elongation at 68°C for 3 min);

5. ~3.8 kb (*A. waldeni*), ~4,5 kb (*P. panini*): Pen_Cyt1065-for and Buce_12S240-rev (annealing and elongation both at 70°C for 4 min together).

PCR products were subsequently sequenced by using PCR primers and internal primers (see Additional file 3: Table S2. PCR primers used to amplify and sequence mt gene fragments). Because of apparent length heteroplasmy at the end of the control regions, we performed nested PCRs on fragment 4 with the primers AcePen_KR_Rep-for and AcePen_Cyt638-rev and on fragment 5 with the primers AcePen_KR_Rep-for and Acewal_KR_Z-rev for *A. waldeni *and Penpan_KR_Z-rev for *P. panini*, respectively (annealing at 56.2°C, elongation at 70°C for 2 min (for sequences of primers see Additional file 3: Table S2. PCR primers used to amplify and sequence mt gene fragments). Another nested PCR was performed on fragment 5 using primers AcePen_KRII-for and AvesDiv_Phe-rev. All these nested PCR products were cloned using the TOPO TA Cloning Kit for Sequencing (Invitrogen) according to manufacturer's instructions. At least 30 clones of each PCR were sequenced.

1,930 bp encompassing the duplicated parts of *Cytb*, tRNA T/rRNA P/*NADH6*/tRNA E and domains I and the first parts of domains II of the CRs of 10 additional individuals were determined by PCR amplification and sequencing the fragments 2, 3, 4, and 5 (cf. Figure 1A).

### Alignments and gene annotation

Transfer RNA genes (tRNA) were identified by their potential secondary structure and anticodon sequence using the tRNAscan-SE Server [49]. tRNA S2 was not found by the server. Thus, we constructed the secondary structure manually using tRNA S2 alignments from other birds. The boundaries of ribosomal RNA genes (rRNA) and the control region were inferred from boundaries of flanking genes under the assumption that there are neither intergenic spacers nor overlaps. Start positions of protein-coding genes preceded by tRNAs were defined by the first potential start codon after the end of the flanking tRNA. Start positions of protein-coding genes preceded by other protein-coding genes were determined by aligning them to other avian mt genomes using the BioEdit Sequence Alignment Editor [50]. This method was also used to verify the boundaries of all other genes. Stop codons of all protein-coding genes were determined according to Slack et al. [34].

### Analyses of sequence data

To infer the pattern of evolution of the duplicated region, we conducted a phylogenetic analysis for 6 individuals per species. The two copies were included separately in each analyses and are designated by an abbreviation of the species name, a unique number for the individual, and a roman letter to distinguish the copies (e.g., Pp-1-I and Pp1-1-II for the two duplicates of individual number 1 of *Penelopides panini*). We conducted separate analyses for the region without the putative Replication Fork Barrier (RFB) and for the putative RFB fragment only (for determination of the RFB region see Figure 1 and text below).

All four datasets (Pp_without_RFB, Pp_RFB_only, Aw_without_RFB, Aw_RFB_only) were aligned in BioEdit [50]. Maximum likelihood (ML) analysis of the dataset was conducted using RAxML version 7.0.3 [51], using GTR+GAMMA+P-Invar model parameters (4 gamma categories). GTR is the only available nucleotide substitution model in RAxML.

To test for gene conversion, we compared (a) the mean p-distance between duplicates within any individual (πdupl) and (b) the mean p-distance between individuals separately for each of the two duplicates (πind). This analysis was performed separately for the inferred Replication Fork Barrier (RFB; 159 bp in *A. waldeni*, 59 bp in *P. panini*; shaded grey in Figure 1B, C) and the remaining non-RFB parts of the duplicates (1,368 bp before and 403 bp after RFB in *A. waldeni*, 1,388 bp before and 483 bp after RFB in *P. panini*; unshaded regions in Figure 1B, C) using Mega 3.1 [52]. Without gene conversion, the two duplicates are assumed to evolve independently after the duplication event, such that πind should be expected to be significantly higher than πdupl. This pattern should disappear in the case of gene conversion, due to homogenization across duplicates.

Additionally, we compiled a dataset including all available hornbill data for the central region of the control region (*Aceros waldeni *[Genbank: HQ834450]; *Penelopides panini *[GenBank: HQ834451]; *Bucorvus leadbeateri *[GenBank: AY027930]; *Tockus erythrorhynchus damarensis *[GenBank: AY027932]; *Tockus erythrorhynchus kempi *[GenBank: AY027927]; *Tockus erythrorhynchus rufirostris *[GenBank: AY027928]; *Tockus leucomelas *[GenBank: AY027931]; and *Tockus monteiri *[GenBank: AY027934]). Sequences from *Grus leucogeranus *[GenBank: AF112371], *Chlamydotis undulata fuertaventurae *[GenBank: AJ544568], and *Thalassarche melanophris *[GenBank: AY158677] served as outgroups. Sequences were aligned using MAFFT version 6 [53], using the iterative refinement method E-INS-i. Maximum likelihood (ML) analysis of the dataset was conducted using RAxML version 7.0.3 [51], using GTR+GAMMA+P-Invar model parameters (4 gamma categories). Support values were estimated by 1,000 bootstrap replicates.

## Authors' contributions

SS designed the study, conducted the lab work, carried out the sequence alignment, performed genetic analysis, and drafted the manuscript. CB participated in designing the study, performed genetic analysis, wrote parts of the methods subsection regarding "Analyses of sequence data", and revised the manuscript. RT supervised and participated in designing the study, supervised data analysis, and critically revised the manuscript. All authors read and approved the final manuscript.

## Supplementary Material

Additional file 1 **Sequence annotation of the mt genome of *A. waldeni*/*P. panini *(as in deposited sequence)**. Click here for file

Additional file 2 **ML-analysis of the central domain of the control region of Bucerotidae**. GTR + G + I model of sequence evolution. Bootstrap support from 1,000 replicates is given at the nodes. Note that published sequences for *Bucorvus leadbeateri *and *Tockus erythrorhynchus *sequences are indistinguishable from one another, which points to the possibility that the published *Bucorvus *sequence originates from a contamination. Click here for file

Additional file 3 **PCR primers used to amplify and sequence mt gene fragments**. Click here for file
