# Supplementary material for: Full mitochondrial genome sequences of two endemic Philippine hornbill species (Aves: Bucerotidae) provide evidence for pervasive mitochondrial DNA recombination
Source: BMC Genomics. 2011 Jan 14;12:35. doi: 10.1186/1471-2164-12-35 (PMC3025957; doi:10.1186/1471-2164-12-35)
Supplement: Additional file 1 — Sequence annotation of the mt genome of A. waldeni/P. panini (as in deposited sequence). [file 1471-2164-12-35-S1.DOC]

Table S1. PCR primers used to amplify and sequence mt gene fragments

| **PCR primers** | **Temp.^a^** | **Primer sequence (5’-3’)** |
| --- | --- | --- |
| fragment 1 (~14 kb) |  |  |
| AcePen_12S_68-for | 58.2 | GTGTAAATGCCCATAGCCCCTTAC |
| AcePen_Cytb250-rev | - | GAAGAGTGAGGCTCCGTTGGCATG |
| AcePen_Cyt1018-rev^b^ | - | GGGTGTTCTACTGGTTGGCTGCC |
| fragment 2 (~1.6 kb) |  |  |
| AcePen_Cytb253-for | 58 | GCCAACGGAGCCTCACTCTTCTTCATCTG |
| AcePen_Glu-rev^b^ | - | ATTTTTAAGGYTTTTCTGTGGTTC |
| fragment 3 (~3.2 kb (*A. waldeni*),  ~3,6 kb (*P. panini*)) |  |  |
| AcePen_Glu-for^b^ | 58 | GCTTTTCTCCAAGGTCTACAGCTC |
| AcePen_Cyt1018-rev^b^ | - | see above |
| fragment 4 (~2.8 kb (*A. waldeni*),  ~3,1 kb (*P. panini*)) |  |  |
| AcePen_KRII-for^b^ | 56 | GGTTTGGCACTGTTGGTTCCCTT |
| AcePen_Glu-rev^b^ | 56 | see above |
| fragment 5 (~3.8 kb (*A. waldeni*),  ~4,5 kb (*P. panini*)) |  |  |
| Pen_Cyt1065-for^b^ | 58 | GTCCATCACCTACTTCACCACCCTCCTC |
| Buce_12S_240-rev | 58 | GACCGCGGTGGCTGGCACAAGATTTACC |
|  |  |  |
| **internal primers (location)** | **Temp.** ^a^ | **Primer sequence (5’-3’)** |
| fragment 1 |  |  |
| AcePen_12S_977-for | 57 | CAAGGTAAGTGTACCGGAAGGTG |
| L2725 (*16S*) | 50 | [24] |
| L3218 (*16S*) | 57 | [24] |
| L3803 (*16S*) | 57 | [24] |
| AcePen_ND1-for | 50.5 | CAATAATCGTACTAAGCGGAAAC |
| L5143 (tGln) | 57 | [24] |
| L5758 (*NADH2*) | 57 | [24] |
| L6335 (Trp) | 55 | [24] |
| AcePen_COI-for | 59.7 | CCCACTGGCCGGCAACTTAGC |
| L7525 (*COI*) | 54.4 | [24] |
| L7987 (*COI*) | 55 | [24] |
| L8386 (*COII*) | 54.4 | [24] |
| L8929 (*COII*) | 57 | [24] |
| AcePen_ATP6-for | 48.5 | CCTACTACCCTACACATTTACCC |
| L9700 (*ATP6*) | 59.7 | [24] |
| L10236 (*COIII*) | 55 | [24] |
| L10635 (*COIII*) | 57 | [24] |
| AcePen_ND4L-for | 58.2 | CCACCCTAATCCCCATCCTCAT |
| L11458 (*ND4L*) | 57 | [24] |
| L12156 (*ND4*) | 57 | [24] |
| AcePen_ND4-for | 58.2 | TACCCCCGCACTCCTCCTAATC |
| L12912 (His) | 57 | [24] |
| L13525 (*NADH5*) | 59.7 | [24] |
| L14080 (*NADH5*) | 54.4 | [24] |
| AcePen_ND5-for | 56.4 | CTAATCCACCGCTCCAGCTCA |
| AcePen_Cytb253-for | 58 | see above |
| fragment 2 |  |  |
| LCytb-AcPe^b^ | 48 | GTAACCCCTCCCCACATC |
| Pen_Cytb969-for^b^ | 59.7 | GATCCTATTCTGAACCCTAACCACCAACCT |
| Thr-L^b^ | 43.2 | [46] |
| fragment 3 |  |  |
| AcePen_KRII-for^b^ | 58 | see above |
| AcePen_KR_Rep-for^b^ | 58 | ATTTTACACTTCCTCTAACTTTTC |
| Acewal_KR_Z-rev^b^ (only *A. waldeni*) | - | GGCGGGTGAAGTATGTTAAAGTCT |
| Penpan_KR_Z-rev^b^ (only *P. panini*) | - | TGTGTTTGGGTTTGTTAGTGTGGC |
| Acewal_KR_Z-for^b^ (only *A. waldeni*) | 58 | GACTTTAACATACTTCACCCGCCG |
| Pen_KR_Z-for^b^ (only *P. panini*) | 58.2 | GCCACACTAACAAACCCAAACACA |
| AcePen_Cyt638-rev^b^ | 54.4 | GGGATTTTGTCGCAGTTGGATG |
| fragment 4 |  |  |
| LCytb-AcPe^b^ | 48 | see above |
| Pen_Cytb969-for^b^ | 59.7 | see above |
| fragment 5 |  |  |
| Thr-L^b^ (only *A. waldeni*) | 43.2 | [46] |
| HBND6L (only *A. waldeni*) | 48.5 | [46] |
| AcePen_Glu-for^b^ | 58 | see above |
| AcePen_KRII-for^b^ | 58 | see above |
| AcePen_KR_Rep-for^b^ | 58 | see above |
| Acewal_KR_Z-rev^b^ (only *A. waldeni*) | - | see above |
| Penpan_KR_Z-rev^b^ (only *P. panini*) | - | see above |
| Acewal_KR_Z-for^b^ (only *A. waldeni*) | 58 | see above |
| Pen_KR_Z-for^b^ (only *P. panini*) | 58.2 | see above |
| AvesDiv_Phe-rev | 48 | CATCTTCAGTGCCATGCTT |
| AcePen_Phe-for | 59.7 | CCAAGATGGCTGCTCGACATGC |

^a^ temperature (in °C) used in sequencing reaction

^b^ primers with two annealing sites due to duplication

- sequencing reaction failed
