# Supplementary material for: Full mitochondrial genome sequences of two endemic Philippine hornbill species (Aves: Bucerotidae) provide evidence for pervasive mitochondrial DNA recombination
Source: BMC Genomics. 2011 Jan 14;12:35. doi: 10.1186/1471-2164-12-35 (PMC3025957; doi:10.1186/1471-2164-12-35)
Supplement: Additional file 2 — ML-analysis of the central domain of the control region of Bucerotidae. GTR + G + I model of sequence evolution. Bootstrap support from 1,000 replicates is given at the nodes. Note that published sequences for Bucorvus leadbeateri and Tockus erythrorhynchus sequences are indistinguishable from one another, which points to the possibility that the published Bucorvus sequence originates from a contamination. [file 1471-2164-12-35-S2.PDF]

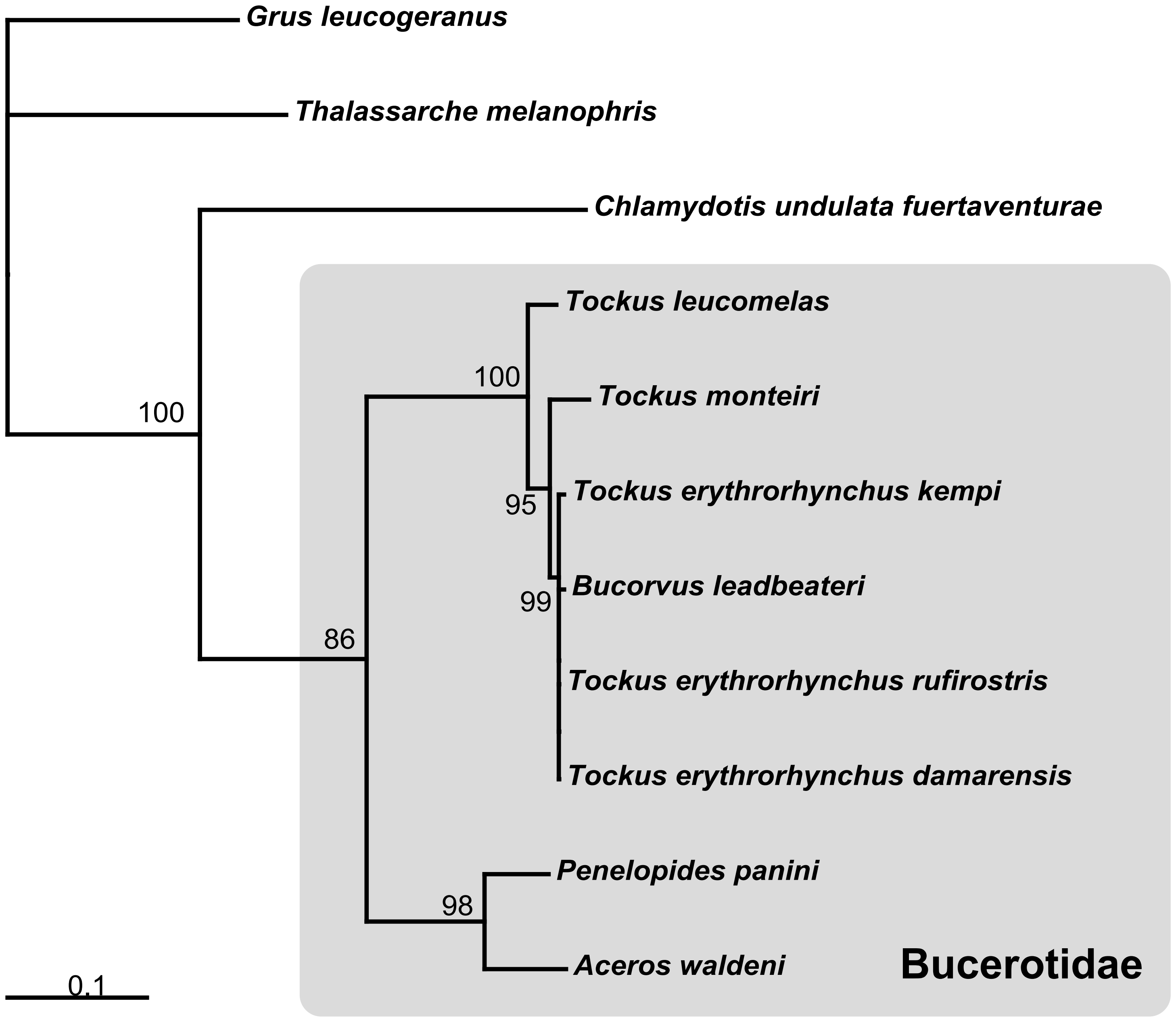

Suppl. Fig. 1: ML-analysis of the central domain of the control region of Bucerotidae under the GTR + G + I model of sequence evolution. Bootstrap support from 1000 replicates is given at the nodes. Note that *Bucorvus leadbeateri* and *Tockus erythrorhynchus* sequences are indistinguishable and therefore, it is highly likely that the *Bucorvus* sequence originates from a contamination.
