# Supplementary material for: Full mitochondrial genome sequences of two endemic Philippine hornbill species (Aves: Bucerotidae) provide evidence for pervasive mitochondrial DNA recombination
Source: BMC Genomics. 2011 Jan 14;12:35. doi: 10.1186/1471-2164-12-35 (PMC3025957; doi:10.1186/1471-2164-12-35)
Supplement: Additional file 3 — PCR primers used to amplify and sequence mt gene fragments. [file 1471-2164-12-35-S3.DOC]

Table S2. Sequence annotation of the mt genome of *A. waldeni*/*P. panini* (as in deposited sequence)

| genes | location | size (bp) ^b^ | size (aa)^c^ | start codon | stop codon | spacer/  overlap^d^ |
| --- | --- | --- | --- | --- | --- | --- |
| tRNA F | 1-71 | 71 |  |  |  |  |
|  |  |  |  |  |  | - |
| *12S* | 72-1044 | 973 |  |  |  |  |
|  |  |  |  |  |  | - |
| tRNA V | 1045-1117 | 73 |  |  |  |  |
|  |  |  |  |  |  | - |
| *16S* | 1118-2718/1118-2715 | 1601/1598 |  |  |  |  |
|  |  |  |  |  |  | - |
| tRNA L | 2719-2792/2716-2789 | 74 |  |  |  |  |
|  |  |  |  |  |  | 12 |
| *NADH1* | 2805-3782/2802-3779 | 978 | 325 | ATG | AGG |  |
|  |  |  |  |  |  | 1 |
| tRNA I | 3781-3853/3778-3850 | 73 |  |  |  |  |
|  |  |  |  |  |  | 12 |
| tRNA Q^a^ | 3936-3866/3933-3863 | 71 |  |  |  |  |
|  |  |  |  |  |  | 1 overlap |
| tRNA M | 3936-4004/3933-4001 | 69 |  |  |  |  |
|  |  |  |  |  |  | - |
| *NADH2* | 4005-5045/4002-5042 | 1041 | 346 | ATA | TAA |  |
|  |  |  |  |  |  | 2 |
| tRNA W | 5045-5118/5042-5116 | 74/75 |  |  |  |  |
|  |  |  |  |  |  | 11 |
| tRNA A^a^ | 5198-5130/5196-5128 | 69 |  |  |  |  |
|  |  |  |  |  |  | 12 |
| tRNA N^a^ | 5285-5211/5283-5209 | 75 |  |  |  |  |
|  |  |  |  |  |  | 2 |
| tRNA C^a^ | 5354-5288/5352-5286 | 67 |  |  |  |  |
|  |  |  |  |  |  | 1 overlap |
| tRNA Y^a^ | 5425-5354/5423-5352 | 72 |  |  |  |  |
|  |  |  |  |  |  | 1 |
| *COI* | 5427-6977/5425-6975 | 1551 | 516 | GTG | AGG |  |
|  |  |  |  |  |  | 6 overlap |
| tRNA S^a^ | 7042-6969/7040-6967 | 74 |  |  |  |  |
|  |  |  |  |  |  | 3 |
| tRNA D | 7046-7114/7044-7112 | 69 |  |  |  |  |
|  |  |  |  |  |  | 1 |
| *COII* | 7116-7799/7114-7797 | 684 | 227 | GTG | TAA |  |
|  |  |  |  |  |  | 4 |
| tRNA K | 7801-7873/7799-7871 | 73 |  |  |  |  |
|  |  |  |  |  |  | 1 |
| *ATP8* | 7875-8039/7873-8037 | 165 | 54 | ATG | TAA |  |
|  |  |  |  |  |  | 7 overlap |
| *ATP6* | 8030-8713/8028-8711 | 684 | 227 | ATG | TAA |  |
|  |  |  |  |  |  | 2 |
| *COIII* | 8713-9496/8711-9494 | 784 | 261 | ATG | T-- |  |
|  |  |  |  |  |  | 1 |
| tRNA G | 9497-9565/9495-9563 | 69 |  |  |  |  |
|  |  |  |  |  |  | - |
| *NADH3* | 9566-9917/9564-9915 | 352 | 116 | ATC | TAA |  |
|  |  |  |  |  |  | 5 |
| tRNA R | 9920-9988/9918-9986 | 69 |  |  |  |  |
|  |  |  |  |  |  | 1 |
| *NADH4L* | 9990-10286/9988-10284 | 297 | 98 | ATG | TAA |  |
|  |  |  |  |  |  | 4 overlap |
| *NADH4* | 10280-11657/10278-11655 | 1378 | 459 | ATG | T-- |  |
|  |  |  |  |  |  | 1 |
| tRNA H | 11658-11726/11656-11724 | 69 |  |  |  |  |
|  |  |  |  |  |  | 1 |
| tRNA S2 | 11728-11793/11726-11791 | 66 |  |  |  |  |
|  |  |  |  |  |  | -/11 |
| tRNA L | 11794-11864/11803-11873 | 71 |  |  |  |  |
|  |  |  |  |  |  | - |
| *NADH5* | 11865-13685/11874-13694 | 1821 | 606 | GTG/ATG | TAA |  |
|  |  |  |  |  |  | 2 |
| *Cytb* | 13685-14827/13694-14836 | 1143 | 380 | ATG | TAG |  |
|  |  |  |  |  |  | 6 |
| tRNA T (1) | 14831-14900/14840-14909 | 70 |  |  |  |  |
|  |  |  |  |  |  | 7 |
| tRNA P (1)^a^ | 14978-14908/14987-14917 | 71 |  |  |  |  |
|  |  |  |  |  |  | 11 |
| *NADH6* (1)^a^ | 15508-14987/15517-14996 | 522 | 173 | ATG | TAA |  |
|  |  |  |  |  |  | 2 |
| tRNA E (1)^a^ | 15583-15511/15592-15520 | 73 |  |  |  |  |
|  |  |  |  |  |  | - |
| CRI | 15584-17684/15593-18082 | 2101/2490 |  |  |  |  |
|  |  |  |  |  |  | - |
| *Cytb* (2) | 17685-18210/18083-18608 | 526 | 174,33 | / | TAG |  |
|  |  |  |  |  |  | 6 |
| tRNA T (2) | 18214-18283/18612-18681 | 70 |  |  |  |  |
|  |  |  |  |  |  | 7 |
| tRNA P (2)^a^ | 18361-18291/18759-18689 | 71 |  |  |  |  |
|  |  |  |  |  |  | 11 |
| *NADH6* (2)^a^ | 18891-18370/19289-18768 | 522 | 173 | ATG | TAA |  |
|  |  |  |  |  |  | 2 |
| tRNA E (2)^a^ | 18966-18894/19292-19364 | 73 |  |  |  |  |
|  |  |  |  |  |  | - |
| CRII | 18967-21657/19365-22737 | 2691/3373 |  |  |  |  |
|  |  |  |  |  |  | - |

^a^ transcribed from light strand

^b^ including start and stop codon (for reasons see [34])

^c^ size of protein-coding genes without stop codon and extra base of *NADH3* (for reasons see [34])

^d^ spacers include stop codons of preceding protein-coding gene, overlaps do not include stop codons (for reasons see [34])
